# Modulation of Glutamate Release by Dexmedetomidine Preserves Dendritic Spines and Alleviates Cognitive Impairment in a Murine Model of Perioperative Neurocognitive Disorder

**DOI:** 10.1007/s12264-025-01518-w

**Published:** 2025-10-17

**Authors:** Yan Zhang, Junzhao Li, Xueju Wang, Zhongyu Zhang, Shuai Long, Chuanyu Edward Li, Yan Liu, John Man Tak Chu, Raymond Chuen-Chung Chang, Gordon Tin-Chun Wong, Yong Zhang

**Affiliations:** 1 State Key Laboratory of Common Mechanism Research for Major Diseases, Institute of Basic Medical Sciences Chinese Academy of Medical Sciences, School of Basic Medicine Peking Union Medical College, Beijing, 100005 China; 2 Neuroscience Center, Chinese Academy of Medical Sciences, Beijing, 100005 China; 3 Department of Anaesthesiology, LKS Faculty of Medicine, The University of Hong Kong, Pokfulam, Hong Kong SAR, China; 4 Neuroscience Research Institute and Department of Neurobiology, School of Basic Medical Sciences, Peking University, Key Laboratory for Neuroscience, Ministry of Education of China and National Health Commission of the People’s Republic of China, Beijing, 100083 China; 5 Department of Obstetrics and Gynecology, Peking University Third Hospital, Beijing, 100191 China; 6 Zhejiang Provincial Key Lab of Addiction Research, The Affiliated Kangning Hospital of Ningbo University, Ningbo, 315201 China; 7 Department of Rehabilitation, Shenzhen Nanshan People’s Hospital, Shenzhen, 518052 China; 8 Laboratory of Neurodegenerative Diseases, School of Biomedical Sciences, LKS Faculty of Medicine, The University of Hong Kong, Hong Kong SAR, China; 9 Department of Anaesthesiology, The University of Hong Kong, Queen Mary Hospital, Hong Kong SAR, China; 10 IDG/McGovern Institute for Brain Research at PKU, Beijing, 100871 China

**Keywords:** Hippocampus, Medial prefrontal cortex, Glutamate, Excitatory amino-acid transporter-2, Excitotoxicity, Dexmedetomidine

## Abstract

Perioperative neurocognitive disorders (PNDs) represent a significant challenge in the perioperative setting, while the pathophysiology of PNDs remains unclear. Utilizing a murine model of abdominal surgery, we found that abnormal glutamatergic neurotransmission in the medial prefrontal cortex (mPFC) and hippocampus contributes to postoperative cognitive impairments. Increases in the frequency of miniature excitatory postsynaptic currents in both the mPFC and CA1 neurons indicate enhanced presynaptic glutamate release while having little effect on inhibitory neurotransmission. Surgery also enhances glutamate release from presynaptic terminals in the Schaffer collateral pathway. In addition, abdominal surgery increased the activation of microglia and astrocytes, elevated central inflammatory markers, and reduced excitatory amino-acid transporter-2 expression. Dexmedetomidine significantly mitigates the postoperative cognitive deficits by reducing inflammation and preserving neuronal structural complexity and dendritic spine stability, likely through inhibiting glutamate release and enhancing its reuptake. These findings advance our understanding of the etiology of PNDs and provide hints for potential intervention.

**Supplementary Information:**

The online version contains supplementary material available at 10.1007/s12264-025-01518-w.

## Introduction

Perioperative neurocognitive disorders (PNDs) are characterized by a range of cognitive impairments, such as memory loss, difficulties in concentration, and impaired executive function, that can arise after surgical procedures [1]. The pathogenesis of PNDs is complex and may involve postoperative neuroinflammation, oxidative stress, autophagy dysfunction, and impaired synaptic function [2–5]. Glutamate, the primary excitatory neurotransmitter in the brain, plays a central role in synaptic transmission and brain function. When released in excess, however, it can lead to excitotoxicity that can disrupt neuronal network function, impair information processing, and ultimately result in neuronal damage or death [6–8]. The inflammatory response elicited by surgical trauma may indirectly affect glutamate release. Inflammatory factors such as interleukin-1β (IL-1β) can activate microglia, which can then affect the function of presynaptic neurons and glutamate release [9, 10]. Excitatory amino-acid transporters (EAATs) are a group of transport proteins responsible for glutamate clearance from the synaptic cleft and maintenance of physiological glutamate levels in the central nervous system (CNS) that negates neuronal overactivation and glutamate excitotoxicity [11]. EAAT2 is the most prominent glutamate transporter in the CNS, and its dysfunction has been associated with various neurodegenerative diseases, such as Alzheimer's disease, amyotrophic lateral sclerosis, and epilepsy [12–14]. The abnormal release or clearance of glutamate may play a significant role in the development of PNDs.

Dexmedetomidine, a selective α2-adrenergic receptor agonist, has been shown to inhibit glutamate release [15–18]. This inhibition is primarily mediated through its action on presynaptic α2-adrenergic receptors. It has been shown that dexmedetomidine can protect neurons from excitatory amino-acid-induced excitotoxicity and injury [19, 20]. Dexmedetomidine can also attenuate lipopolysaccharide (LPS)-induced neuroinflammation and enhance astrocytic glutamine metabolism, reducing the availability of glutamine as a neurotoxic precursor to glutamate synthesis [21, 22]. In addition, dexmedetomidine has been shown to increase the expression of EAAT3, which plays a critical role in glutamate reuptake in neurons and glia [23].

The medial prefrontal cortex (mPFC) and hippocampus are critical regions involved in memory and cognition [24]. A recent study has identified two parallel mPFC-amygdala pathways that are implicated in memory consolidation and extinction in the postoperative brain [25]. However, the specific mechanisms of glutamate neurotransmission in the mPFC and/or hippocampus remain relatively unexplored in the context of PNDs. In this study, we used a murine surgical model and applied a comprehensive set of techniques, including behavioral analysis, biochemical assays, electrophysiology, and two-photon *in vivo* imaging for morphological assessment to further characterize glutamatergic neurotransmission in these two regions. We hypothesized that adverse effects in the postoperative brain from neuroinflammation may be alleviated by α2 receptor-mediated inhibition of glutamate release. Our findings have demonstrated that both pre- and postsynaptic abnormalities contribute to the cognitive deficits after laparotomy and advanced our understanding of the neural mechanisms underlying PNDs.

All the abbreviations used in this article are listed in Table 1.

**Table 1:** List of abbreviations.

AMPA	α-Amino-3-hydroxy-5-methyl-4-isoxazolepropionic acid
APs	Action potentials
CNS	Central nervous system
EAATs	Excitatory amino acid transporters
EAAT2	Excitatory amino acid transporter-2
eEPSCs	Evoked excitatory postsynaptic currents
EMP	Elevated plus-maze
GAPDH	Glyceraldehyde 3-phosphate dehydrogenase
GFAP	Glial fibrillary acidic protein
IL-1β	Interleukin-1β
IL-6	Interleukin-6
IL-10	Interleukin-10
LPS	Lipopolysaccharide
LTP	Long-term potentiation
mEPSCs	Miniature excitatory postsynaptic currents
mIPSCs	Miniature inhibitory postsynaptic currents
mPFC	Medial prefrontal cortex
MWM	Morris water maze
NMDA	N-methyl-D-aspartate
NOR	Novel object recognition
OFT	Open field test
PBS	Phosphate-buffered saline
PNDs	Postoperative neurocognitive disorders
ROIs	Regions of interest
RT-qPCR	Real-time quantitative polymerase chain reaction
TNF-α	Tumor necrosis factor alpha

## Materials and Methods

### Animals

All animal studies and experimental procedures were approved by the Animal Care and Use Committee of the Animal Facility at Peking University Health Science Center. Twelve-week-old C57BL/6N mice were obtained from the Department of Laboratory Animal Sciences at Peking University Health Science Center (Beijing, China). The animals were housed in a controlled environment with a stable temperature (22 ± 2 °C), humidity (50% ± 10%), and a 12-h light/dark cycle (lights on at 08:00). They had free access to food and water throughout the study.

### Surgical Model

An abdominal surgical model was used following our previously established protocols [26]. Each mouse was placed in a plexiglass induction chamber, and anesthesia was induced with 5% sevoflurane and maintained with 3% sevoflurane inhalation at a flow rate of 0.8 L/min. A ~2 cm longitudinal skin incision was made along the linea alba. Approximately 10 cm of intestines were exteriorized, gently rubbed for 2 min, and exposed to open air for an additional minute before being replaced into the abdominal cavity. The muscle and skin layers were sutured separately. The entire surgical procedure was completed within 30 min. The sham group received only sevoflurane at the same concentrations and for the same duration, while the wild-type group was not exposed to sevoflurane. In the treatment groups, 50 μg/kg dexmedetomidine dissolved in 0.9% saline or 0.9% saline alone was administered by intraperitoneal injection at 0, 2, and 4 h after surgery.

### Open Field Test (OFT)

The OFT was applied under dim lighting conditions (50 lux) and was video recorded for subsequent analysis using Ethovision XT software. Each mouse was placed in the center of a white acrylic behavior box measuring 40 cm × 40 cm × 40 cm and allowed to move freely for 5 min. The total distance traveled and the time spent in the center area were measured and analyzed [27]. The box was cleaned with 75% alcohol after each test to eliminate any residual odor.

### Novel Object Recognition (NOR) Test

During the habituation phase, each mouse was allowed to explore freely for 5 min. On the second day, during the familiarization phase, two identical objects (A) were placed in diagonally opposite quadrants of the testing arena. Each mouse was placed in the center and allowed to explore for 10 min. One hour later, one of the objects was replaced with a new object (B), differing in material, size, color, and shape. Each mouse was then returned to the arena to explore the new object (B) and the familiar object (A) for another 10 min, constituting the short-term memory test. Twenty-four hours later, object (B) was replaced with object (C) for the long-term memory test. An interaction was recorded when the mouse's mouth, nose, or front paws directly touched the object or approached within 2 cm of it. The preference index was calculated as the new object exploration time divided by the total exploration time (new object exploration time + old object exploration time) multiplied by 100% [28].

### Y-maze Test

The Y-maze test was used to assess spatial working memory by recording spontaneous alternation behavior. The Y-maze consisted of three arms, each measuring 35 cm ×10 cm in width with a 20 cm high wall (denoted A, B, and C), positioned at 120° from each other. Each mouse was placed at the center of the symmetrical Y-maze and allowed to freely explore each of the three arms for 8 min. An arm entry was defined as the entry of all four limbs and the tail into one arm of the maze. Unrepeated consecutive arm entries (e.g., ABC, BCA, CAB) were defined as a successful triad combination. The percentage of spontaneous alternation was calculated as (number of successful triad combinations) / (total number of arm entries-2) × 100 [29].

### Elevated Plus Maze (EPM) Test

The EPM device consisted of two open arms, each measuring 30 cm × 5 cm with no sidewalls, and two closed arms of similar length and width but with a 15-cm high side wall, connected by a central 5 cm square area. The device was placed 50 cm above the ground. The test was conducted under dim lighting conditions (50 lux). Each mouse was placed in the central area of the device, with its head facing an open arm, and allowed to explore freely for 5 min. The time spent and the number of entries into the open and closed arms were video-recorded and analyzed using Ethovision XT software [30]. After each test, the device was cleaned with 75% alcohol to eliminate any influence from residual odors.

### Morris Water Maze (MWM) Test

The maze consisted of a water tank 1.5 m in diameter, containing water at between 19 °C and 22 °C. Tests were conducted under dim lighting conditions at 50 lux. The escape platform was removed for a 1-min free-swimming test to allow animals to adapt to the maze environment and to check for substantial differences in baseline exploratory behaviors. After the free-swimming test, the animals were dried with a hairdryer. Training began 30 min later, with the escape platform placed 1 cm below the water surface. Each animal was trained 5 times per day for 1 min, beginning from semi-random starting positions. If an animal failed to locate the platform within 1 min, it would be gently guided towards it. Training commenced 7 days after surgery for the test of learning and memory. The probe test was then conducted 24 h after the last training session, when each animal was tested for 1 min without the escape platform, starting from the quadrant opposite the target platform. For the memory retrieval test, water maze training was conducted before surgery, and the probe test was applied seven days after surgery. Time spent in the target zones, number of entries, and escape latency were recorded [31].

### Immunofluorescent Staining

The mice were euthanized with CO_2_ after testing and were then perfused with 0.9% saline to flush out the blood from the brain, followed by perfusion with 4% paraformaldehyde. The brains were collected by decapitation and placed in 4% paraformaldehyde overnight for fixation. Subsequently, the brains were dehydrated by immersion in 20% and then 30% sucrose solutions until they sank, indicating full dehydration. The dehydrated brains were cut at 30 µm on a cryostat. For the reconstruction of pyramidal neurons in the mPFC, the sections were further fixed by immersion in 4% paraformaldehyde following a 0.1% biocytin injection during electrophysiological recording. The sections were then immunostained. Non-specific binding sites were first blocked with 5% bovine serum albumin at room temperature for 1 h and then incubated overnight at 4°C with the following primary antibodies: anti-Iba-1 (1:300, Abcam, AB178847), anti-GFAP (1:500, Cell Signaling Technology, CST 3670S), and anti-EAAT2 (1:500, Proteintech, 22515-1-AP). The sections were rinsed with PBS-Triton X-100 and incubated for 2 h at room temperature with fluorochrome-conjugated secondary antibodies. These were anti-mouse Alexa Fluor 594 (1:1000, Abcam, ab150120) for GFAP detection, anti-rabbit Alexa Fluor 488 (1:1000, Abcam, ab150081) for detecting Iba-1, anti-rabbit Alexa Fluor 488 (1:500, Abcam, ab150081) for EAAT2, and streptavidin (1:500, Alexa Fluor 647-conjugated, Invitrogen, S21374). Following this secondary antibody incubation, the sections were mounted on slides using a mounting medium containing DAPI. Images were captured under a laser confocal microscope (Leica TCS-SP8 DIVE) equipped with a 20× objective lens at a resolution of 1024 × 1024 pixels, with Z-stack images taken at a step size of 1 µm. Images were analyzed using ImageJ software (National Institutes of Health, USA). Structural reconstruction of astrocytes and neurons, as well as Sholl analysis, was conducted using the Simple Neurite Tracer plugin in ImageJ. All quantitative analyses were applied using a minimum of three images per animal, collected from four independent experiments [32].

### Whole-Cell Patch-Clamp Recording

Recordings were made from pyramidal neurons in Layer V of the prelimbic cortex and the CA1 region of the hippocampus. Each brain was cut on a vibratome in a dissection medium containing (in mmol/L): 75 sucrose, 87 NaCl, 3 KCl, 1.5 CaCl₂, 1.3 MgSO₄, 1 NaH₂PO₄, 26 NaHCO₃, and 20 D-glucose, at pH 7.4–7.5 and osmolality 313 mOsm. The slices were then placed in a recording chamber and perfused with artificial cerebrospinal fluid (aCSF) saturated with 95% O₂ and 5% CO₂, containing (in mmol/L): 124 NaCl, 3 KCl, 2 CaCl₂, 1.3 MgSO₄, 1 NaH₂PO₄, 26 NaHCO₃, and 20 D-glucose, at pH 7.4–7.5 and osmolality 295 mOsm.

For recording miniature excitatory postsynaptic currents (mEPSCs), miniature inhibitory PSCs (mIPSCs), and evoked excitatory PSCs (eEPSCs), the pipette was filled with an internal solution containing (in mmol/L): 110 CsMeSO_3_, 15 CsCl, 10 HEPES, 0.5 EGTA, 4 ATP-Mg, 0.3 Tris-GTP, 5 sodium phosphocreatine, 4 QX-314, and 0.1% biocytin, at pH 7.2 and osmolality 275 ± 5 mOsm. For current-clamp recordings of action potentials (APs), the pipette was filled with a solution containing (in mmol/L): 120 potassium D- gluconate, 10 KCl, 4 Mg-ATP, 0.3 Na-GTP, 5 Na phosphocreatine, 2 EGTA, and 10 HEPES, at pH 7.2 and osmolality 275 ± 5 mOsm. Tetrodotoxin (1 μmol/L) was added to the aCSF for recording mEPSCs (holding potential at -70 mV) and mIPSCs (holding potential at 0 mV). The paired pulse ratio (PPR) test involves applying two consecutive pulses at different time intervals, specifically at 50, 100, and 200 ms. AMPAR (α-amino-3-hydroxy-5-methyl-4-isoxazolepropionic acid receptor ) and NMDAR (N-methyl-D-aspartate receptor) currents were recorded at -70 mV and +40 mV, respectively.

For long-term potentiation (LTP) recording, the extracellular recording electrode was placed in the stratum radiatum of the CA1 region. The field excitatory postsynaptic potential (fEPSP) was evoked by a glass electrode positioned in the CA3 region. The aCSF-filled recording electrode (125 NaCl, 2.5 KCl, 1.25 NaH_2_PO_4_·2H_2_O, 25 NaHCO_3_, 10 D-Glucose, 2 CaCl_2_·2H_2_O, and 1.5 MgSO_4_, saturated with 95% O_2_ and 5% CO_2_, pH 7.2–7.4) had a stimulation resistance of ~500–700 kΩ and a recording resistance of ~1.0 MΩ. The stimulating electrode was placed 200-400 μm away from the recording electrode. The baseline stimulation intensity was set to elicit 40%–50% of the maximum response amplitude. After stabilization of the response, baseline recordings were taken over 30 min, and the slope fluctuation was maintained within 10%. LTP was then induced using theta-burst stimulation (TBS). Each session consisted of 10 bursts, with 2 sessions delivered at 20-s intervals; each burst contained 4 pulses at 100 Hz, with 200 ms between bursts. Following TBS, single test pulses were delivered for 60 min to monitor the response [33].

Data were acquired using a MultiClamp 700B amplifier and Clampfit software. Data were sampled at 10 kHz and filtered at 2 kHz. The pipette resistance was 4–6 MΩ. Data were excluded if the series resistance exceeded 20 MΩ, changed by more than 20% during recording, or if the baseline was unstable. Data were analyzed using Clampfit and Origin software [34, 35].

### **Real-time Quantitative Polymerase Chain Reaction (RT-qPCR)**

Freshly-isolated tissues were preserved in RNA keeper-ICE tissue transfer buffer (Vazyme, China). Total RNA from the mPFC tissue was extracted using the FastPure Cell/Tissue Total RNA Isolation Kit (Vazyme, China) under RNase-free conditions, following the manufacturer’s protocol. The extracted mRNA was then reverse-transcribed into cDNA using the HiScript® III RT SuperMix for qPCR (+gDNA wiper) (Vazyme, China). For amplification, ChamQ Universal SYBR qPCR Master Mix (Vazyme, China), primers, and templates were added to the reaction system. Inflammatory cytokines were assessed by real-time PCR. The primer sequences used were as follows: (1) IL-1ß, forward: CCTCCTTGCCTCTGATGG, reverse: AGTGCTGCCTAATGTCCC; (2) IL-10, forward: CCAAGCCTTATCGGAAATGA, reverse: TTCTCACCCAGGGAATTCAA; (3) glyceraldehyde 3-phosphate dehydrogenase (GAPDH), forward: ATTCAACGGCACAGTCAA, reverse: CTCGCTCCTGGAAGATGG. The expression level of the target RNA was normalized to GAPDH using the 2^-ΔΔCt^ method.

### **Craniotomy and Adeno-associated Virus (AAV) Injection**

Each mouse was fixed in a stereotaxic apparatus, and anesthesia was induced using 5% sevoflurane, maintained at 3% during the procedure. A 3 mm × 3 mm cranial window was created above the barrel cortex for two-photon imaging. We also conveniently perform the injection of AAV through this opening. The virus AAV2/9-CaMKIIα-CSSP-dsRed2-2E4 (5.28 × 1012 viral genomes per ml) was injected at 2-3 sites, each site receiving 100 nl at a depth of ~300 μm. After injection, a glass coverslip was placed over the cranial window and sealed with dental cement (C&B Metabond, Parkell). A custom-made metal head plate was then attached to the skull. Each mouse was housed individually for three weeks before undergoing further procedures and imaging [36].

### **Two-photon Imaging**

Two-photon imaging was performed on awake mice using a Leica two-photon microscope (TCS-SP8 DIVE). Baseline images were captured through the cranial window before the surgery. The apical dendrites of layer 2/3 pyramidal neurons in the barrel cortex were imaged using a 20×/1.0 NA water-immersion objective. dsRed was excited at 910 nm with ~100 mW of power delivered to the back aperture of the objective. Red fluorescence signals were separated using ET605/70m dichroic mirrors and filters. Images were acquired at a resolution of 1024×1024 pixels with a voxel size of 0.18 µm in the x and y dimensions, and a z-step of 1 µm. Representative images were median or Gaussian blur-filtered and contrast-enhanced.

### **Analysis of in-vivo Two-Photon Imaging Data**

All spine dynamics and intensity analyses were applied using custom-written software in Igor Pro (WaveMetrics). Using the raw image stacks from the dsRed channel, each dendrite was traced to generate a dendritic backbone and radius, providing information on geometrical structure for subsequent dendritic spine analysis. This was mainly accomplished by a modified version of the Simple Neurite Tracer plugin in FIJI. Each spine was then visually identified at each time point and manually marked at the endpoint. Spines connected to the dendritic backbone following the brightest linear path were identified, and by measuring the distribution distance along the dendritic backbone between each spine, corresponding spines in images from different time points were semi-automatically identified. Finally, the connections of each spine to the dendritic backbone and the correspondence of spines across different time points were manually checked one by one. To ensure the accuracy of the analysis, spines had to protrude more than 4 pixels (>0.72 μm) from the dendritic backbone and be primarily parallel to the imaging plane to be included in the statistics. Spine density, addition, elimination, and turnover ratio were analyzed using the same dendritic tracings and spines. Intensity analysis was conducted on spines that were visually identifiable at all time points. Spines were divided into three distinct, non-overlapping regions of interest (ROIs): spine, shaft, and background ROIs. Each ROI is extended in three dimensions, encompassing the area above and below the optimal imaging plane. All data were analyzed using the same methods as in our previous publication [37].

### **Statistical Analysis**

All statistical analyses were applied using Prism 10.1 (GraphPad Software, USA). Data are presented as the mean ± SEM and were initially examined for normal distribution using the Shapiro-Wilk normality test. For data that were normally distributed with homogeneous variance, parametric tests were used. Depending on the number of factors, an unpaired Student’s *t*-test, one-way ANOVA, or two-way ANOVA was applied, followed by Tukey’s test or Bonferroni’s test for *post hoc* analysis. A *P*-value <0.05 was considered statistically significant.

## Results

### Surgical Trauma Impairs Short-Term and Long-Term Memory, While Showing No Effects on Anxiety Levels

**Fig. 1:**
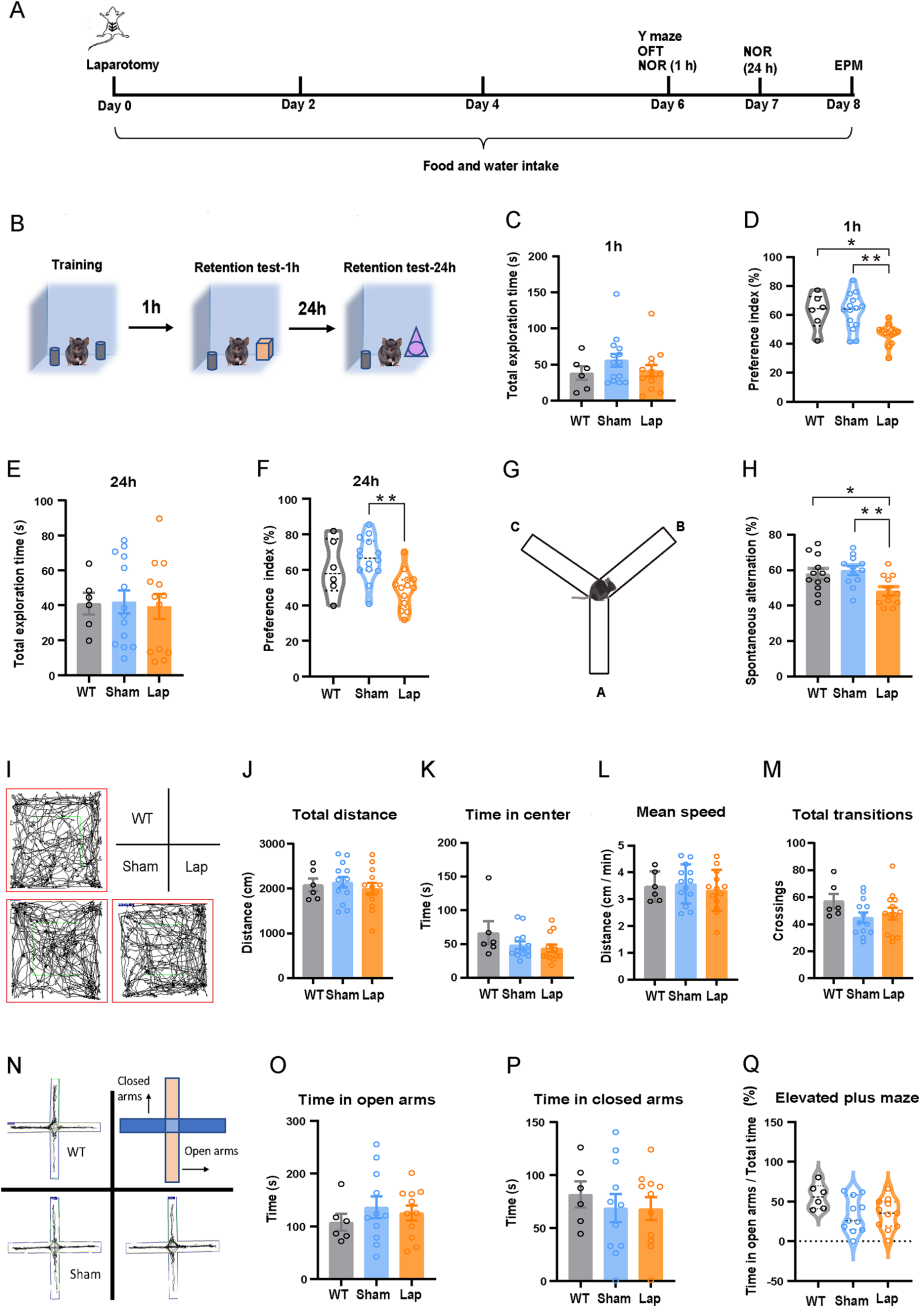
Laparotomy impairs working memory while showing no effects on motor function and anxiety behaviors. **A** Timeline of the experimental design. **B** Diagram of the setup for the NOR test. **C**-**F** Performance in the NOR test. *n =* 6–13 mice. **G**, **H** Performance in the Y-maze test. *n =* 12 mice. **I**-**M** Locomotor paths and performance indices in the OFT. **N**–**Q** Locomotor paths and performance indices in the EPM test. Data are presented as the mean ± SEM. *P < 0.05; **P < 0.01, by one-way ANOVA with Tukey’s multiple comparisons test for **C**–**F**, **H**, **J**-**M**, and **O**–**Q**. *WT* wild type, *Lap* laparotomy, *OFT* open field test, *NOR* novel object recognition, *EPM* elevated plus-maze.

The experimental timeline for investigating the effects of surgery on memory and behavior is illustrated in Fig. 1A. Throughout the experimental period, food and water consumption remained stable across all groups (Supplementary Fig. S1A, B). Mice were subjected to the novel object recognition (NOR) test (Fig. 1B), a relatively efficient and low-stress task for memory assessment. The laparotomy group showed a significant reduction in the preference index in both the 1-h short-term memory test (Fig. 1C, D) and the 24-h long-term memory test (Fig. 1E, F). Subsequently, the Y-maze test was applied to further investigate working memory (Fig. 1G), and a decreased spontaneous alternation ratio was found in the laparotomy group compared to the sham and WT groups (Fig. 1H). As shown in Fig. 1I–M, there were no significant differences in the distance traveled, time spent in the center of the area, mean speed, or total transitions among groups as assessed by the OFT. In addition, in the EPM test, no significant differences were detected in the time spent in the open or closed arms (Fig. 1N–Q), which further confirmed that laparotomy did not induce anxiety-like behaviors. Subsequently, the MWM test results showed that surgery did not affect the acquisition (Supplementary Fig. S1C–F) or retrieval of the acquired spatial memory (Supplementary Fig. S1C, G–I). Together, these findings suggest that surgical trauma specifically impairs working memory without inducing anxiety behaviors.

### Surgical Trauma Increases the Activation of Microglia and Astrocytes in the mPFC and Subregions of the Hippocampus

**Fig. 2:**
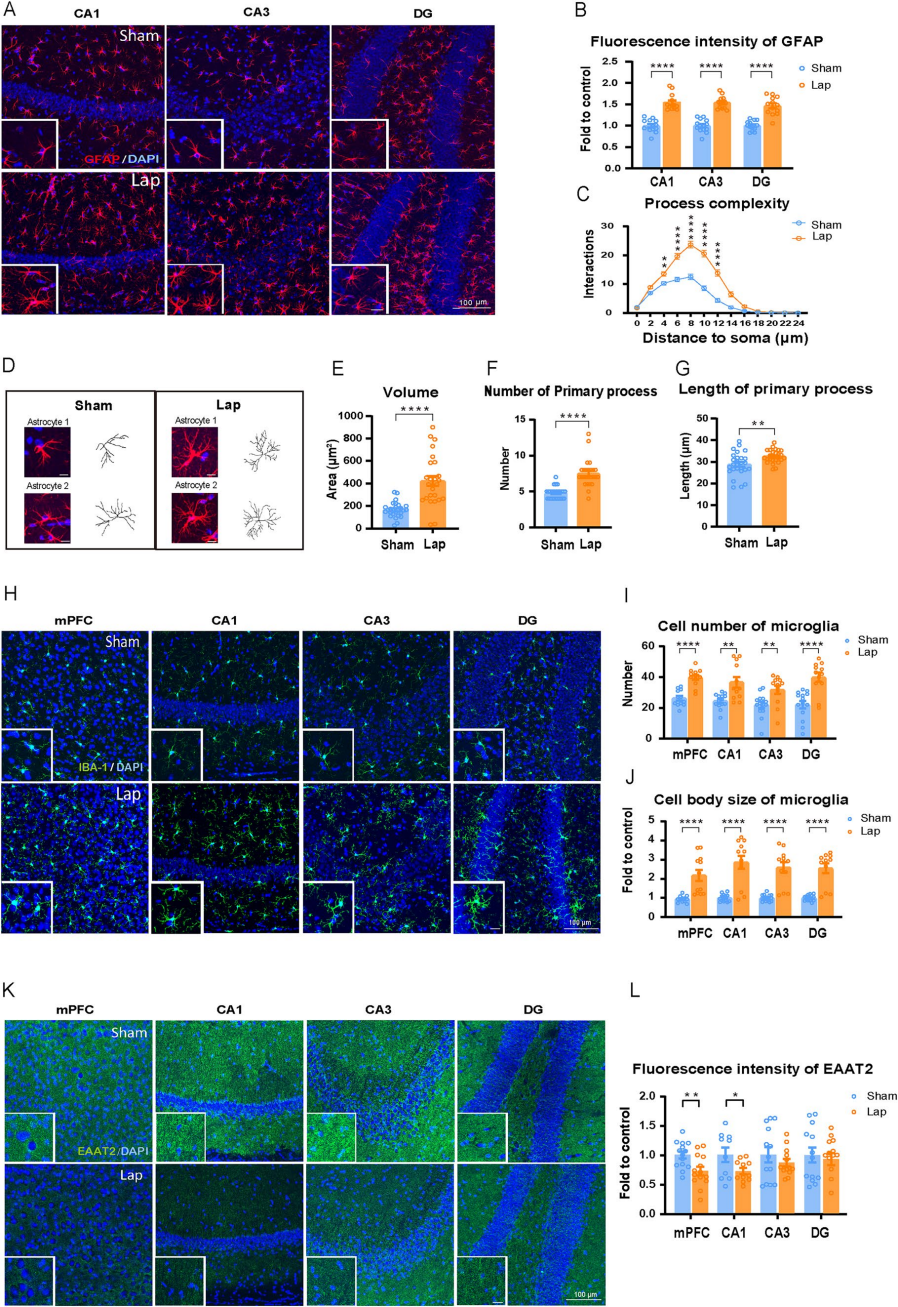
Laparotomy increases the activation of microglia and astrocytes and decreases the expression of EAAT2. **A** Confocal images taken with a 20× objective lens showing GFAP-positive astrocytes (red) and DAPI (blue) in the CA1, CA3, and DG regions of the hippocampus. Scale bar = 100 μm (for expanded images, scale bar = 20 μm). **B** Quantification of GFAP fluorescence intensity. **C**,** E**–**G** Morphological analysis of reconstructed astrocytes. **D** Representative images of reconstructed astrocytes. Astrocyte reconstruction performed using the Simple Neurite Tracer (SNT) plugin in ImageJ. **H** Iba-1-positive microglia (green) and DAPI (blue) in the mPFC, and the CA1, CA3, and DG regions of the hippocampus. Scale bars = 100 μm or 20 μm. **I** Numbers of Iba-1-positive microglia in the mPFC and hippocampal sub-regions, analyzed using ImageJ software. **J** Measurement of cell body size of Iba-1-positive microglia. *n =* 4 mice per group. **K** Representative images showing EAAT2 (green) and DAPI (blue) in the mPFC and sub-regions in the hippocampus. Scale bars = 100 μm or 20 μm. **L** Quantification of EAAT2 fluorescence intensity. *n =* 4 mice per group. Data are represented as mean ± SEM. *P < 0.05; **P < 0.01; ****P < 0.0001, by unpaired Student’s *t*-test for **B**, **E**–**G**, **I**, **J** and** L**; two-way ANOVA with Bonferroni’s multiple comparisons test for **C**. *Lap* laparotomy.

Glial fibrillary acidic protein (GFAP) staining showed a significant increase in fluorescence intensity in astrocytes within the hippocampal regions CA1, CA3, and dentate gyrus (DG) after surgery (Fig. 2A, B). Further analysis showed a marked increase in the complexity of astrocytic processes within a 2-24 µm radius around the cell body (Fig. 2C), suggesting that surgery induced greater morphological complexity in astrocytes. Structural reconstruction of astrocytes in the CA1 region also demonstrated that surgery significantly increased the astrocyte volume, as well as the number and length of primary processes (Fig. 2D–G). There was also a significant increase in the number and size of ionized Ca^2+^- binding adapter molecule 1-positive microglia after surgery (Fig. 2H–J). These results indicate morphological remodeling and functional alterations in glial cells after surgery, which are often associated with neuroinflammation or neurodegenerative processes.

### Surgical Trauma Reduces EAAT2 Expression and Increases the Release of Inflammatory Factors

EAAT2s are abundantly expressed in astrocytes in the hippocampus, cortex, and cerebellum, and play a critical role in regulating synaptic transmission and maintaining the stability of the CNS [38]. EAAT2s were co-stained with GFAP and DAPI (Supplementary Fig. S2A). Surgery significantly reduced EAAT2 fluorescence intensity in the mPFC and CA1 regions compared to the sham group, while no significant differences were found in the CA3 and DG (Fig. 2K, L). This reduction in EAAT2 expression indicates a weakened capacity to clear glutamate, which may lead to elevated glutamate levels in the synaptic cleft and an increased risk of excitotoxicity.

In response to infection, injury, or other stimuli, activated astrocytes and microglia can secrete IL-1β, a pro-inflammatory cytokine [39], which plays a crucial role in immune responses, particularly in initiating and sustaining inflammation. In our study, IL-1β mRNA expression significantly increased in the mPFC and CA1 regions after surgery compared to the sham group (Supplementary Fig. S3A, C). IL-10, an anti-inflammatory cytokine, acts to suppress excessive inflammatory responses by inhibiting the production and release of pro-inflammatory cytokines such as IL-1β, tumor necrosis factor alpha (TNF-α), and IL-6, thereby preventing overactivation of the immune system and protecting tissues from inflammation-induced damage [40]. As shown in Supplementary Fig. S3B, a significant increase in IL-10 expression was found in the mPFC following surgery, which may serve to balance the pro-inflammatory response and prevent excessive inflammation. The concurrent increase in both inflammatory and anti-inflammatory cytokines indicates the presence of neuroinflammation after surgery.

### Surgical Trauma Increases Pre-Synaptic Glutamate Release Without Altering Intrinsic Excitability or Inhibitory Neurotransmission

APs reflect neuronal excitability and synaptic transmission. Seven days after surgery, we recorded the voltage changes of pyramidal neurons in layer V of the mPFC following the injection of currents ranging from 50 to 400 pA (Supplementary Fig. S3E–G). Compared to the sham group, no significant changes were found in the frequency of APs, depolarization threshold, or input resistance (Supplementary Fig. S3H–J). Similarly, afterhyperpolarization amplitude, peak amplitude, and AP half-width also remained unchanged (Supplementary Fig. S3K–M). These measurements were repeated 14 days post-surgery (Supplementary Fig. S3N–P), and no changes were found compared to the sham group (Supplementary Fig. S3Q–V). These results indicate that surgery does not alter the intrinsic excitability of layer V neurons in the mPFC.

**Fig. 3:**
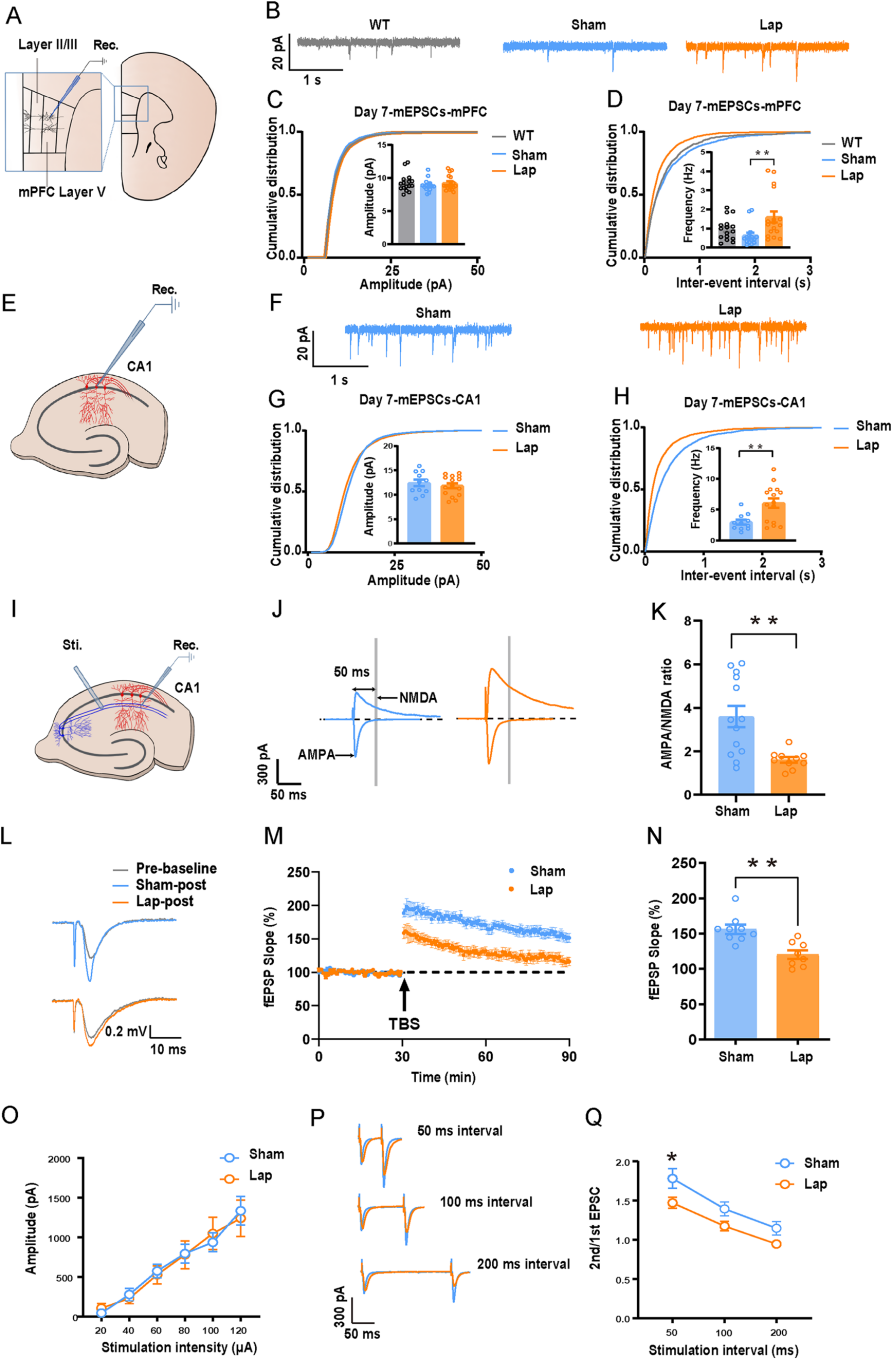
Laparotomy increases presynaptic glutamate release and impairs LTP. **A** Schematic of recording location L5 of neurons in the mPFC (blue box indicates recording location). **B** Schematic of mEPSC traces in the mPFC 7 days after laparotomy, *n =* 15–17 neurons.** C**,** D** Cumulative probability of the frequency and amplitude of mEPSCs in the mPFC 7 days post laparotomy, *n =* 15–17 neurons. **E** Schematic of recording location in CA1 neurons of the hippocampus. **F** Schematic of the mEPSC traces in CA1 7 days after laparotomy. **G**,** H** Cumulative probability of the frequency and amplitude of mEPSCs in CA1 7 days post laparotomy, *n =* 11–15 neurons. **I** Schematic of the locations of stimulation and recording electrodes in the hippocampus. **J** Schematic of evoked AMPAR and NMDAR component currents.** K** AMPA/NMDA ratio, *n =* 10–13 neurons. **L** Schematic of LTP traces.** M** fEPSP slope 30 min before and 60 min after TBS delivery.** N** Bar graphs of the average percentage changes in the fEPSP slope 50–60 min after TBS delivery (*n =* 8–9 slices from 3 to 4 mice per group). **O** Input/output current analysis, *n =* 10 neurons**. P** Representative EPSC traces with stimulus intervals of 50, 100, and 200 ms. **Q** 2nd/1st EPSC at different stimulation intervals, *n =* 14–15 neurons. Data are presented as the mean ± SEM. **P < 0.01, by unpaired Student’s *t*-test for **G**,** H**, **K**, and **N**; one-way ANOVA with Tukey’s multiple comparisons test for **C** and **D**; two-way ANOVA with Bonferroni’s multiple comparisons test for **O** and** Q**. *WT* wild type, *Lap* laparotomy.

mEPSCs are triggered by the activation of postsynaptic receptors by glutamate release from individual synaptic vesicles, and the frequency and amplitude of mEPSCs reflect presynaptic glutamate release and the number or function of postsynaptic receptors. Similarly, the frequency and amplitude of mIPSCs are associated with GABA release and the number or function of postsynaptic GABA receptors. Memory consolidation involves the transfer of memory from the hippocampus to the cortex. Next, we assessed excitatory and inhibitory neurotransmission by recording mEPSCs/mIPSCs in layer V neurons of the mPFC and CA1. The frequency of mEPSCs increased significantly after surgery compared to sham or WT groups, while the amplitude remained unchanged (Fig. 3B–H). This increase in mEPSC frequency persisted at 14 days post-surgery (Supplementary Fig. S4A–F), indicating the enhancement in mEPSC frequency is long-lasting. In contrast, no significant changes were recorded in either the frequency or the amplitude of mIPSCs in the mPFC or CA1 (Supplementary Fig. S4G–L). These findings suggest that surgery enhances glutamatergic release in both the mPFC and the hippocampus while showing little effect on GABAergic neurotransmission.

The Schaffer collateral pathway is a critical component of hippocampal circuitry, particularly between the CA3 and CA1 regions. Schaffer collaterals project to synapses on the dendrites of pyramidal neurons in CA1. eEPSCs represent the collective synaptic input and are commonly used to study synaptic behavior under stimulation conditions. We found a decrease in the α-amino-3-hydroxy-5-methyl-4-isoxazolepropionic acid/N-methyl-D-aspartate (AMPA/NMDA) ratio (Fig. 3I–K), suggesting altered postsynaptic receptor function. Hippocampal LTP is widely recognized as a fundamental cellular mechanism for spatial learning and memory, so we examined the LTP changes following laparotomy. Our results showed a reliable induction of hippocampal CA1 LTP (Fig. 3M), but this LTP was significantly impaired in the surgery group (Fig. 3N). In examining the PPR, no differences were detected between groups in eEPSC amplitudes at various stimulation intensities, indicating that the efficiency of synaptic transmission was not significantly altered by surgery (Fig. 3O). However, we found that under two consecutive stimulations at a 50-ms interval, the PPR value in the surgical group significantly decreased (Fig. 3P, Q), which typically indicates an increased probability of neurotransmitter release from the presynaptic neurons. These results suggest that while laparotomy did not alter the intrinsic excitability of mPFC neurons, it significantly increased presynaptic glutamate release and enhanced postsynaptic NMDAR currents. These changes may lead to excessive Ca^2+^ influx, which contributes to excitotoxicity, cellular damage, and potentially neuronal death.

### Dexmedetomidine Reduces the Inflammatory Response of Microglia and Astrocytes and Improves Postoperative Memory

**Fig. 4:**
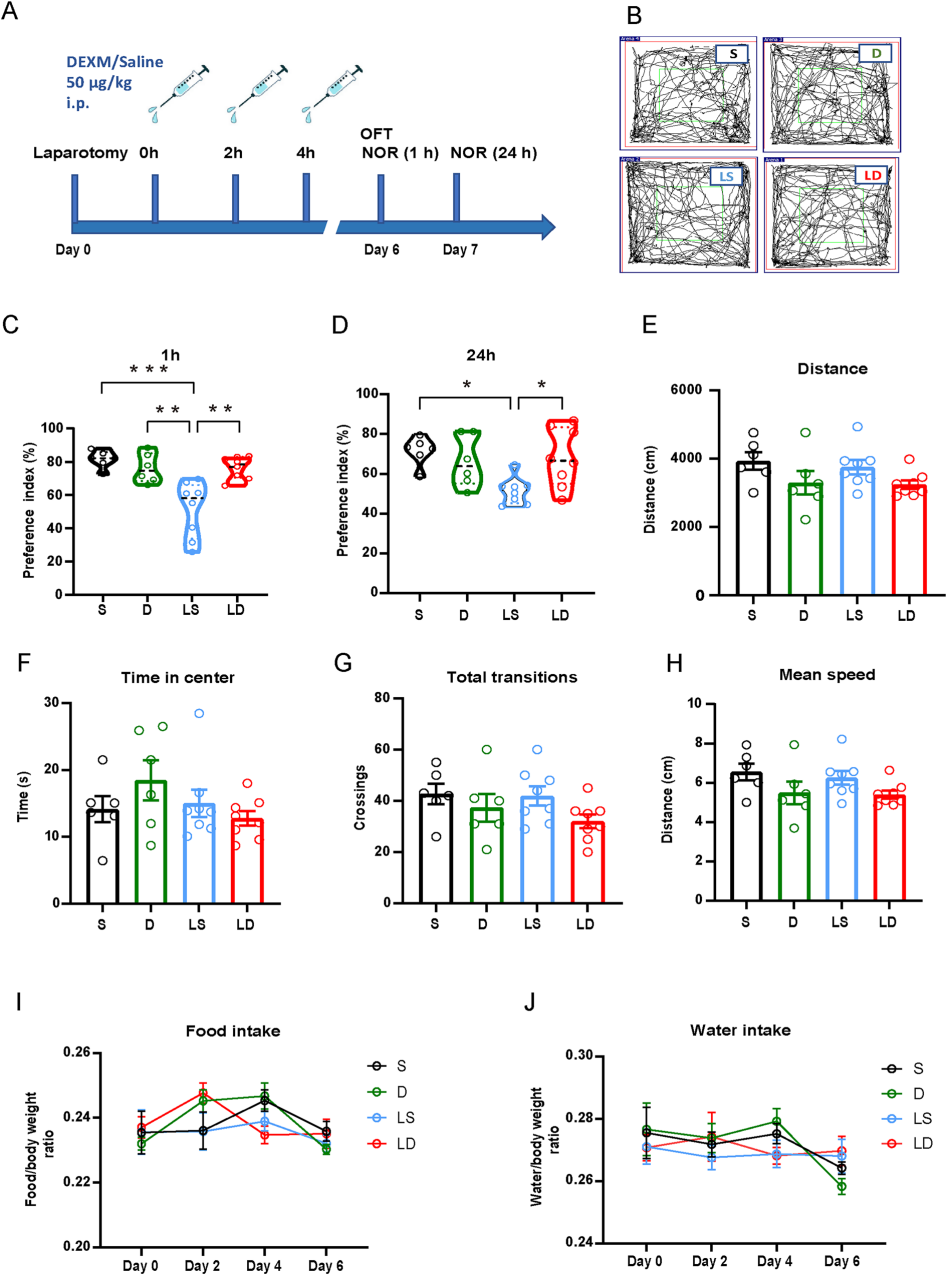
Dexmedetomidine treatment alleviates the memory deficiency without affecting motor function and anxiety behavior. **A** Timeline of the experiment design. B Locomotor paths in the OFT. **C**,** D** Performance in the NOR test, *n =* 6–8 mice. **E**–**H** Performance in the OFT.** I**, **J** Food and water intake normalized to the body weight. Data are presented as the mean ± SEM. *P < 0.05; **P < 0.01; ***P < 0.001, by one-way ANOVA with Tukey’s multiple comparisons test for **C**–**H**; two-way ANOVA with Bonferroni’s multiple comparisons test for **I** and **J**. *S* saline, *D* dexmedetomidine, *LS* laparotomy + saline, *LD* laparotomy + dexmedetomidine.

**Fig. 5:**
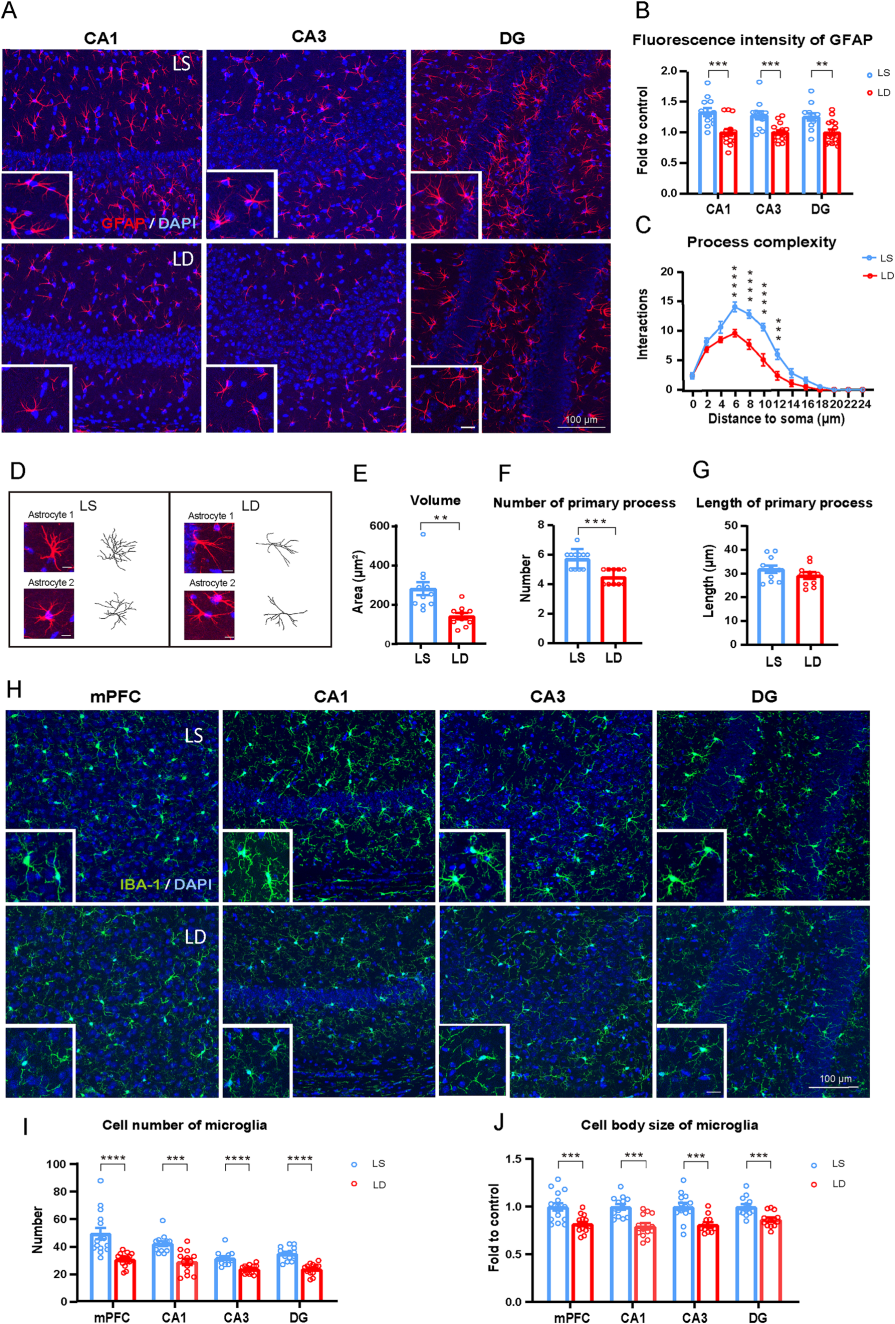
Dexmedetomidine decreases the activation of microglia and astrocytes after treatment. **A** Confocal images taken with a 20× objective lens showing GPAP-positive astrocytes (red) and DAPI (blue) in the CA1, CA3, and DG regions of the hippocampus. Scale bars = 100 μm or 20μm. **B** Quantification of GFAP fluorescence intensity after treatment. **C**, **E**–**G** Morphological analysis of reconstructed astrocytes after treatment. **D** Representative images of reconstructed astrocytes after treatment. Astrocytes reconstructed using the SNT plugin in ImageJ. **H** Confocal images showing Iba-1-positive microglia (green) and DAPI (blue) after treatment in the mPFC and sub-regions of the hippocampus. Scale bars = 100 μm or 20 μm. **I** Cell numbers of Iba-1-positive microglia, analyzed using ImageJ. **J** Cell body size of Iba-1-positive microglia. *n =* 4 mice per group. Data are presented as mean ± SEM. **P < 0.01; ***P < 0.001; ****P < 0.0001, by unpaired Student’s t-test for** B**, **E**–**G**, **I** and **J**; two-way ANOVA with Bonferroni’s multiple comparisons test for **C.** *LS* laparotomy + saline, *LD* laparotomy + dexmedetomidine.

Dexmedetomidine, a selective α2-adrenergic receptor agonist, has been shown to inhibit glutamate release and was used to further explore the role of glutamate release in laparotomy-induced memory impairment. The mice were divided into four groups: saline injection (S), dexmedetomidine injection (D), laparotomy plus saline injection (LS), and laparotomy plus dexmedetomidine injection (LD). An equal volume of saline or 50 μg/kg dexmedetomidine was administered *via* intraperitoneal injection at different time points (Fig. 4A). On the seventh day post-surgery, we applied OFT and NOR tests. Dexmedetomidine significantly increased the preference index both at 1 and 24 h post-surgery compared to the LS group (Fig. 4C, D). In addition, dexmedetomidine did not affect the distance traveled, time spent in the center, total transitions, or mean speed in the OFT (Fig. 4B, E–H). We continuously monitored each mouse's food and water intake over the 7 days and found no significant differences among the four groups (Fig. 4I, J). These results indicate that dexmedetomidine can prevent the decline in memory performance after surgery without affecting locomotor or anxiety levels. To explore the underlying mechanisms, we next examined the effects of dexmedetomidine on microglia and astrocyte activation in the mPFC and hippocampal subregions post-surgery. Through staining and structural reconstruction, we found that dexmedetomidine reduced the fluorescence intensity, process complexity, and cell volume, as well as the number and length of primary astrocytic processes, compared to the LS group (Fig. 5A–G). Similarly, our analysis of microglia in these areas revealed that dexmedetomidine significantly reduced both the number and cell body size of microglia (Fig. 5H–J). In addition, we found that dexmedetomidine significantly decreased the IL-1β level in the mPFC (Supplementary Fig. S3W, X)

### Dexmedetomidine Significantly Inhibits Presynaptic Glutamate Release, Preserves Overall Neuronal Complexity, and Upregulates EAAT2 Expression

**Fig. 6:**
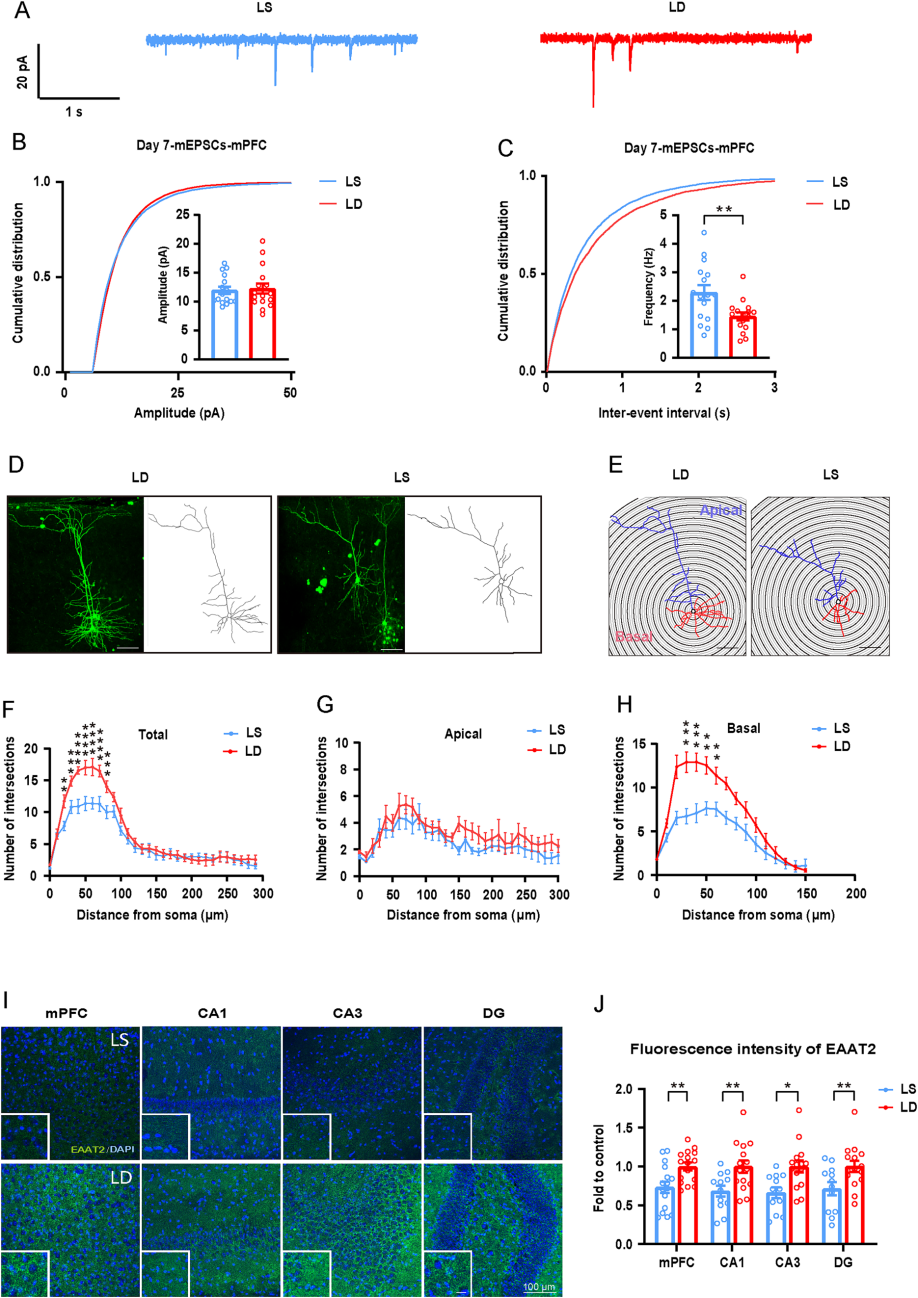
Dexmedetomidine treatment decreases glutamate release and improves dendrite branching complexity in the mPFC after laparotomy. **A** Representative mEPSC traces 7 days after laparotomy following saline or dexmedetomidine treatment. **B**, **C** Cumulative probability of the frequency and amplitude of mEPSCs 7 days after treatment, *n =* 16 neurons. **D** Schematic and reconstruction of streptavidin-stained pyramidal neurons following biocytin injection, Scale bar = 100 μm. E Sholl intersections with varying radial circles. Scale bar = 100 μm. **F**–**H** Sholl intersections of total, apical, and basal dendrites, *n =* 11 neurons. I Representative images of EAAT2 (green) and DAPI (blue) in the mPFC and hippocampal sub-regions. Scale bar = 100 μm or 20 μm. **J** Quantification of EAAT2 fluorescence, *n =* 4 mice per group. Data are presented as the mean ± SEM. *P < 0.05; **P < 0.01; ***P < 0.001; ****P < 0.0001, by unpaired Student’s *t*-test for **B**, **C**, and **J**; two-way ANOVA with Bonferroni’s multiple comparisons test for **F**–**H**. *LS* laparotomy + saline, *LD* laparotomy + dexmedetomidine.

To assess the effects of dexmedetomidine on glutamate neurotransmission, we recorded from the saline and dexmedetomidine treatment groups at 7 days post-surgery. Compared to the LS group, dexmedetomidine significantly reduced the frequency of mEPSCs without affecting their amplitude (Fig. 6A–C). To correlate the electrophysiological properties with neuronal morphology, 0.1% biocytin was added to the internal solution of the recording electrodes. After recording, brain slices were fixed and stained using a streptavidin-conjugated fluorophore, which allowed detailed visualization and structural reconstruction of the recorded neurons. We found that dexmedetomidine significantly improved the complexity of basal and total dendritic branches (Fig. 6D–H). These results indicate that dexmedetomidine not only reduces presynaptic glutamate release but also enhances the structural complexity of dendrites. Based on our findings regarding dexmedetomidine's effects on glutamate release, we sought to investigate how it influences glutamate uptake. We examined the expression of EAAT2 and found that dexmedetomidine significantly increased the expression of EAAT2 post-surgery (Fig. 6I, J) compared to the LS group. This increase in EAAT2 likely contributes to more efficient glutamate clearance from the synaptic cleft, which could reduce the risk of excitotoxicity and promote a healthier balance of excitatory signaling in the brain.

### Dexmedetomidine Reduces Surgery-Induced Dendritic Spine Loss in the Early Postoperative Period

**Fig. 7:**
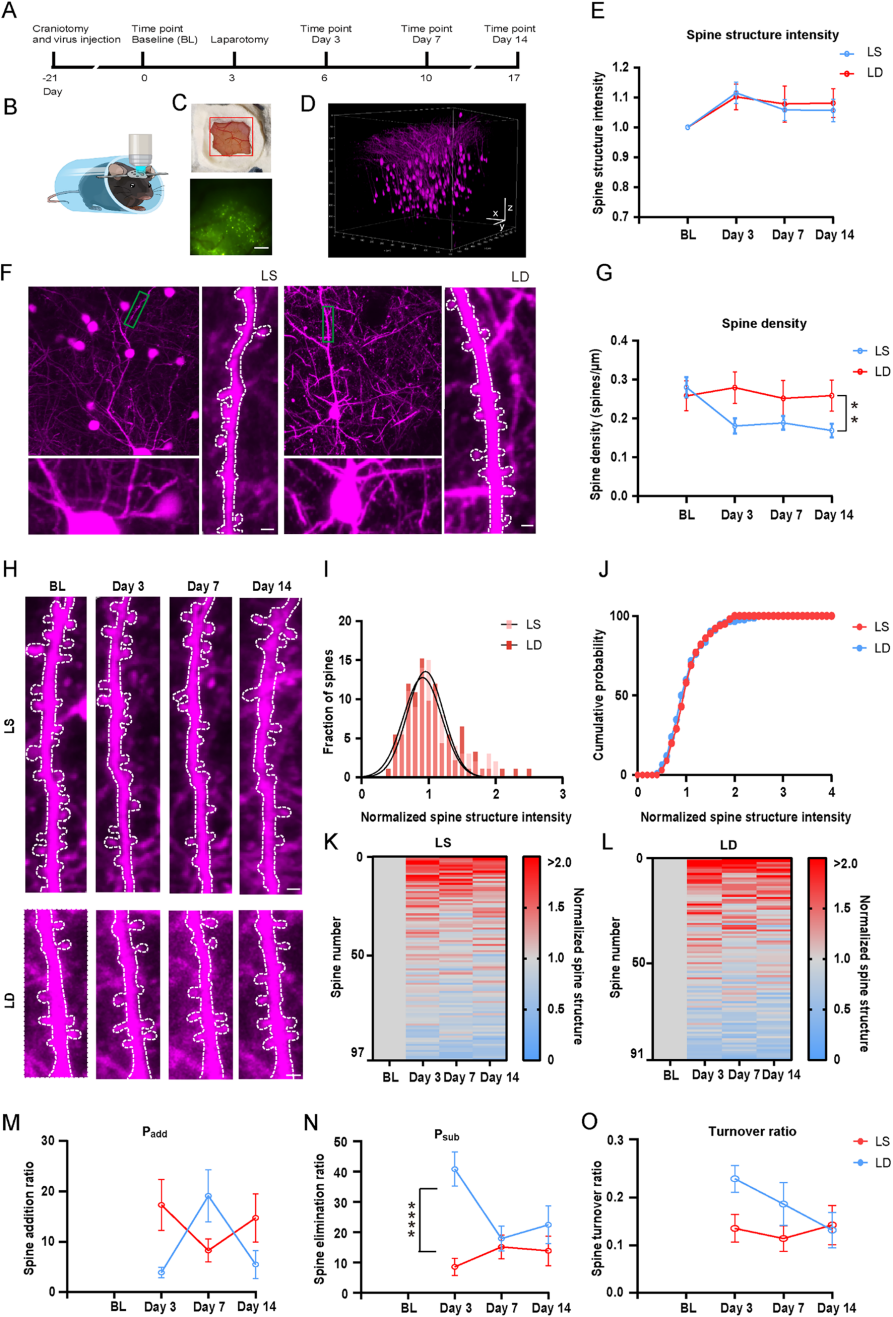
Dexmedetomidine reverses the reduction in synaptic density after laparotomy. **A** Experimental timeline. **B** Schematic of the craniotomy procedure, virus injection, laparotomy, and two-photon imaging. **C** Representative example of the craniotomy window used for imaging and representative time-lapse *in vivo* two-photon image of apical dendrites from layer 2/3 pyramidal neurons. Scale bar = 200 μm. **D** Three-dimensional reconstruction of signals in the barrel cortex of a mouse. Scale bars: x = y = z = 100 μm. **E** Average spine structure intensity normalized to baseline at 3, 7, and 14 days. **F** Representative locations of apical dendrites. Scale bars = 2 μm. **G** Changes in spine density over a period following treatment. **H** Schematic showing spine addition or elimination at 3, 7, and 14 days. Scale bars = 2 μm.** I** Fraction of spines present 7 days after laparotomy. **J** Cumulative probability distribution of spine presence 7 days after laparotomy. **K, L** Heat map of the spine structure intensity following treatment (*LS* group: 100 spines; *LD* group: 93 spines). **M** Ratio of spine addition. **N** Ratio of spine elimination. **O** Spine turnover ratio. Data are presented as the mean ± SEM. **P < 0.01; ****P < 0.0001, by two-way ANOVA with Bonferroni’s multiple comparisons test for **E**, **G**, **M**-**O**. *LS* group: 13 dendrites, 5 mice; *LD* group: 19 dendrites, 6 mice. *LS* laparotomy + saline, *LD* laparotomy + dexmedetomidine, *BL* baseline.

The density and morphology of spines are closely related to an individual's cognitive abilities. Higher spine density is typically associated with enhanced learning capacity and memory function, while a reduction in spine density or abnormalities in spine morphology may lead to a cognitive decline and are often implicated in neurodegenerative diseases. To explore how surgery affects spine dynamics and the potential protective effects of dexmedetomidine, we applied two-photon imaging technology, which enables high-resolution, real-time dynamic imaging, and long-term observation in live animals with lower phototoxicity and photobleaching. For imaging, we used blood vessels to continuously locate the same location and apply XYZ imaging (Fig. 7A–D) as indicated in the schematic diagram. Laparotomy led to a significant reduction in spine density three days post-surgery, which persisted up to 14 days post-surgery (Fig. 7G, blue line). In contrast, the spine density in the LD group remained relatively stable after surgery and was significantly higher than that in the LS group (Fig. 7G, red line). Analysis of spine turnover ratios revealed that the LD group exhibited a significantly lower spine elimination ratio than the LS group (Fig. 7N), suggesting that dexmedetomidine may reverse surgery-induced spine loss by stabilizing existing dendritic spines. Changes in spine size were also assessed: the average spine size of stable spines in both the LS and LD groups showed no significant changes after surgery compared to baseline (Fig. 7E). Taking advantage of the spatiotemporal resolution of *in vivo* imaging, we evaluated individual spine dynamics and found that spine size was quite dynamic across imaging sessions (Fig. 7K, L). However, the distribution of spine size changes was similar in the LS and LD groups 7 days after surgery (Fig. 7I), and the cumulative distribution of spine structure intensity also exhibited no notable differences between the two groups (Fig. 7J). Taken together, while dexmedetomidine treatment did not significantly affect spine size, it demonstrated a protective effect against surgery-induced spine loss.

## Discussion

Our findings revealed an increase in presynaptic glutamate release across several brain regions following surgery, accompanied by cognitive impairment and neuroinflammation. The highly selective α2-adrenergic receptor agonist dexmedetomidine not only reduces presynaptic glutamate release but also attenuates neuroinflammation, maintains dendritic branching complexity, and minimizes spine loss, which helps in preserving baseline cognitive performance after surgery. Accumulating data demonstrate a wide-ranging anti-inflammatory and immunomodulatory effect *via* its actions on immune cells [41] as well as having effects on other pathways. Xiang *et al.* showed that the α7nAChR-mediated cholinergic anti-inflammatory pathway is required for the anti-inflammatory effect of dexmedetomidine after endotoxin injection [42]. Other studies provide support that dexmedetomidine may exert anti-inflammatory effects *via* modulating glial activation, significantly downregulating the expression of inflammatory mediators such as TNF-α, IL-6, and NF-κB [43, 44]. Intrathecal administration of dexmedetomidine suppresses the activation of microglia and astrocytes in the spinal dorsal horn of rats with complete Freund’s adjuvant-induced monoarthritis [45]. Since in this model of peripheral trauma, induced neuroinflammation is likely to involve both central and peripheral inflammatory pathways, it is difficult to ascertain from the current data whether the peripheral or central anti-inflammatory effect predominates. We know that the peripherally administered non-steroidal anti-inflammatory drug ibuprofen can reduce neuroinflammation in an LPS-induced inflammation [46]. The q-PCR from this study demonstrated a reduction in IL-1β expression in the mPFC following dexmedetomidine administration, implying a central anti-inflammatory effect.

The primary glutamate receptors include AMPA receptors, NMDA receptors, and kainate receptors [47]. Glutamatergic neurons are closely linked to cognitive function and learning and memory by regulating neuronal excitability and synaptic plasticity [48]. Clinically, up to 20%–50% of elderly patients exhibit some degree of cognitive impairment within a week after surgery [49]. In our study, mice demonstrated significant cognitive impairment in the NOR test 7 days post-surgery, mimicking human patient conditions. Currently, research in the field of PNDs associated with glutamate transmission predominantly applies molecular biochemical techniques, which have limitations in assessing changes in the glutamatergic system under physiological conditions. We used the whole-cell patch-clamp technique, which allows for the study of neurotransmitter release and ion channel activation while preserving neuronal activity as much as possible, and found that surgery significantly increased the presynaptic glutamate release without altering the number of postsynaptic receptors in both the hippocampus and the cortex. Previous studies have shown that a reduction in AMPAR number is associated with various neurodegenerative diseases, such as Alzheimer’s disease or Parkinson’s disease [50, 51], in which decreased AMPAR expression is thought to directly contribute to cognitive decline. While a significant change in the number of post-operative AMPARs was not found, there were significant abnormalities in presynaptic glutamate release. Excessive glutamate release can overstimulate glutamate receptors, especially NMDARs. This overactivation can lead to a large influx of Ca^2^⁺ into neurons, triggering a series of harmful intracellular reactions such as oxidative stress and mitochondrial dysfunction, ultimately resulting in neuronal damage or death and cognitive impairments [52]. We found a decreased AMPA/NMDA ratio in the hippocampus. This ratio represents the relative proportion of fast, short-term synaptic transmission mediated by AMPARs to the slower, long-term synaptic transmission mediated by NMDARs [53, 54]. A decrease in the AMPA/NMDA ratio may indicate a reduction in synaptic strength or plasticity, which is usually associated with synaptic weakening, abnormal regulation of synaptic plasticity, or changes in neural circuit function. Combined with our previous findings of postoperative mitochondrial dysfunction [26], this suggests that postoperative excitotoxicity may be driven by excessive glutamate release.

Abnormal glutamate release is closely related to neuroinflammation. Microglia and astrocytes play a crucial role in neuroinflammation [55], but there is comparatively less information available regarding the direct effects of dexmedetomidine on these cell types. Astrocytes and microglia express distinct adrenergic receptor subtypes: astrocytes predominantly express α2a and microglia primarily express β2 adrenergic receptors under resting conditions [56]. Notably, microglial adrenergic receptor expression undergoes dynamic regulation during inflammation, switching from β2-adrenergic receptors to α2A-adrenergic receptors under proinflammatory conditions such as LPS treatment [57]. It has been shown that α2 activation in astrocytes can modulate neuronal activity. For instance, Gaidin *et al*. demonstrated that astrocytic α2-adrenergic receptor stimulation triggers GABA release *via* Giβγ subunit-dependent signaling, leading to neuronal suppression [58]. This study is among the first reports of dexmedetomidine influencing presynaptic glutamate release dynamics in the context of postsurgical neuroinflammation. Our study found widespread activation of microglia and astrocytes after surgery, characterized by increased cell volume and process complexity, both of which indicate the presence of neuroinflammation. In our model, we also found an increase in the inflammatory factor IL-1β. Previous studies have shown that activated glial cells can trigger or maintain inflammatory responses by releasing inflammatory factors such as IL-1β and IL-6 and increasing presynaptic glutamate release, which can further enhance neuronal excitability and exacerbate synaptic dysfunction [59]. In addition, astrocytes can influence glutamate levels in the synaptic cleft by regulating the function of glutamate transporters. The excitatory amino-acid transporter family is a group of important membrane transport proteins responsible for regulating glutamate levels, comprising five main subtypes: EAAT1 (GLAST), EAAT2 (GLT-1), EAAT3 (EAAC1), EAAT4, and EAAT5. EAAT3 is primarily expressed in neurons, where it is involved in the clearance of glutamate at the postsynaptic membrane and is responsible for the reuptake and reuse of glutamate by neurons themselves [60]. Previous research using recombinant adeno-associated virus-mediated shRNA to knock down the expression of SLC1A1/EAAT3 in the hippocampus of adult male mice has shown that downregulation of EAAT3 can exacerbate LPS-induced postoperative cognitive impairment [61]. EAAT2 is predominantly expressed in astrocytes and is the main glutamate transporter in the cortex and hippocampus, responsible for ~90% of glutamate clearance in the synaptic cleft, preventing its excessive accumulation and thereby avoiding neuronal hyperexcitability and neurotoxicity. In the hippocampus, EAAT2’s function is critical for learning and memory processes [38]. In epilepsy models, EAAT2 plays a crucial role in preventing excitotoxicity. Upregulating EAAT2 has been shown to reduce spontaneous recurrent seizures and provide neuroprotective effects [62]. The relationship between EAAT2 and the development of POCD remains unclear. Our study found widespread downregulation of EAAT2 expression after surgery, indicating that glutamate clearance may be impaired, finally leading to cognitive impairment.

The activation of α2 receptors leads to a decrease in intracellular cyclic adenosine monophosphate levels, causing Ca^2+^ channels to close, which reduces Ca^2+^ influx and inhibits the release of glutamate from presynaptic neurons [63]. Our study found that the use of dexmedetomidine postoperatively can suppress glutamate release from presynaptic neurons, increase the expression of EAAT2, decrease neuroinflammation, and improve cognitive function. In addition, dexmedetomidine exerted positive effects on postoperative neuronal structure and morphology. Given that structure is the foundation of function, increased structural complexity was seen in the neurons. Complex synaptic networks confer greater plasticity and adaptability in the CNS. Two-photon microscopy enabled longer-term imaging of dendritic spines, thus allowing the observation of dynamic changes in dendritic spines during disease progression and treatment. Dexmedetomidine can significantly inhibit the loss of dendritic spines in the early stages of the disease. The use of this *in vivo* imaging technology to study the changes in dendritic spines during the immediate postoperative period has not been previously reported. However, there is insufficient data in this study to determine whether the beneficial effects of dexmedetomidine are primarily due to its anti-inflammatory effect or its inhibitory effect on glutamate release, which warrants further investigations in the future.

## Conclusions

In the context of postoperative neuroinflammation, we were able to correlate behavioral, molecular, electrophysiological, and structural findings that suggest increased presynaptic glutamate release resulting in a reduction in synaptic strength or plasticity with reduced dendritic spine numbers. We demonstrated the benefits of alpha 2 receptor agonism in reducing these changes and the overall inflammatory response, with improved cognitive performance. While separately some aspects of these results acquired using different techniques have been reported elsewhere, this study was able to integrate them in a single model with *in vivo* imaging under physiological conditions to highlight the functional consequences of the changes.

## Supplementary Information

Below is the link to the electronic supplementary material.

Supplementary file1 (PDF 1095 kb)

## Data Availability

The data that support the findings of this study are available from the corresponding author upon reasonable request.
