# Supplementary material for: Modulation of Glutamate Release by Dexmedetomidine Preserves Dendritic Spines and Alleviates Cognitive Impairment in a Murine Model of Perioperative Neurocognitive Disorder
Source: Neurosci Bull. 2025 Oct 17;42(3):505–24. doi: 10.1007/s12264-025-01518-w (PMC12950140; doi:10.1007/s12264-025-01518-w)
Supplement: Supplementary file 1 — Supplementary file1 (PDF 1095 kb) [file 12264_2025_1518_MOESM1_ESM.pdf]

Supplementary Figures and Figure Legends

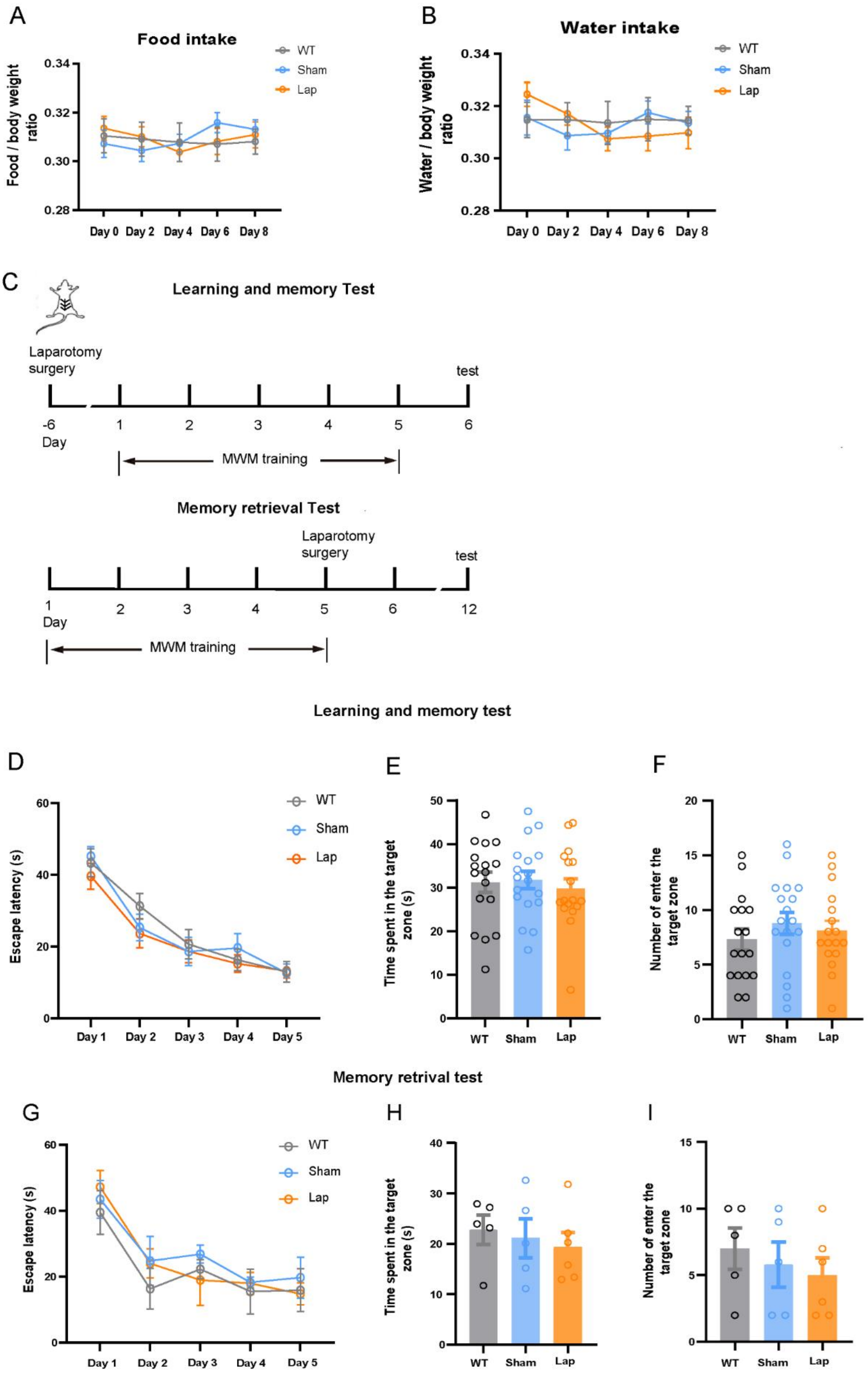

Fig. S1 Laparotomy did not affect the learning, storage, and retrieval of spatial memory. A, B Food and water intake normalized to the body weight. *n* = 6-13 mice**.** **C** Timeline of experiment design in MWM. **D** Learning curve of escape latency in the learning and memory test, *n* = 17-18. **E** Time spent in target zone on the testing day, *n* = 17-18. **F** Number of entries in the target zone on the testing day, *n* = 17-18. **G** Learning curve of the escape latency prior to laparotomy in the memory retrieval test, *n* = 5-6. **H** Time spent in target zone on retrieval day, *n* = 5-6. **I** Numbers of entries in the target zone in the memory retrieval test, *n* = 5-6. Data are presented as mean ± SEM. By one-way ANOVA with Tukey's multiple comparisons test for **E**, **F**, **H**, and **I**; two-way ANOVA with Bonferroni's multiple comparisons test in **A**, **B**, **D**, and **G**. *WT* wild type, *Lap* laparotomy, *MWM*, Morris water maze.

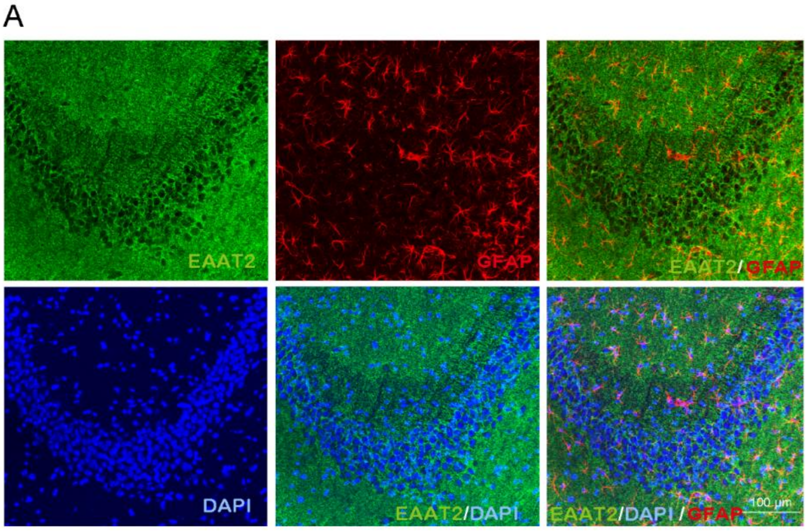

Fig. S2 Schematic diagram of co-staining of the EAAT2/GFAP and DAPI. Scale bar = 100 μm.

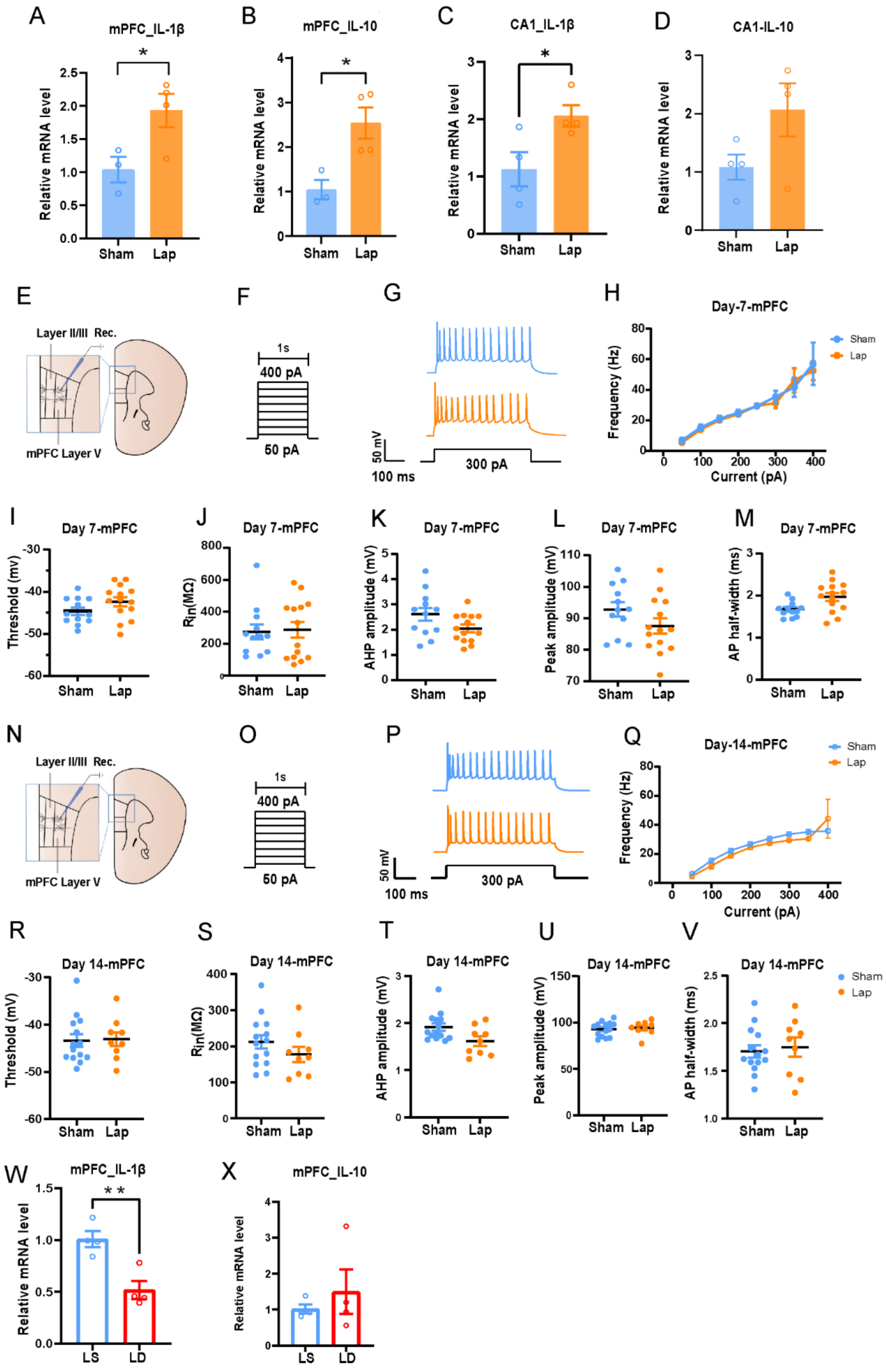
A
mPFC_IL-1β
Relative mRNA level
Sham
Lap
B
mPFC_IL-10
C
CA1_IL-1β
D
CA1-IL-10
E
Layer II/III Rec.
mPFC Layer V
F
1s
400 pA
50 pA
G
50 mV
100 ms
300 pA
H
Day-7-mPFC
Frequency (Hz)
Current (pA)
I
Day 7-mPFC
Threshold (mV)
J
Rin(MΩ)
K
AHP amplitude (mV)
L
Peak amplitude (mV)
M
AP half-width (ms)
N
O
P
Q
Day-14-mPFC
R
Day 14-mPFC
S
T
U
V
W
mPFC_IL-1β
LS
LD
X
mPFC_IL-10

Fig. S3 Laparotomy increased the level of inflammatory cytokines but did not significantly influence intrinsic excitability or inhibitory neuronal transmissions. A-D Relative mRNA levels of IL-1β and IL-10 in the mPFC and CA1. n = 3-4 mice. **E** Schematic diagram of recording position in L5 neurons of mPFC. **F** Schematic diagram of injected currents ranging from 50 pA to 400 pA, in steps of 50 pA. **G** AP spikes with the injected current of 300 pA. **H** Frequency of spikes of mPFC neurons 7 days after surgery, n = 12-14 neurons. **I-M** Depolarization threshold, input resistance, AHP amplitude, peak amplitude, and half-width of mPFC neurons 7 days after laparotomy. n = 12-14 neurons. **N** Schematic diagram of recording position in L5 neurons of mPFC. **O** Frequency with different current injection (from 50 pA to 400 pA, in 50 pA steps). **P** AP spikes with the injected current of 300 pA. **Q** Frequency of spikes of mPFC neurons 14 days after surgery, n = 9-14 neurons. **R-V** Depolarization threshold, input resistance, AHP amplitude, peak amplitude, and half-width of mPFC neurons 14 days after laparotomy, n = 9-14 neurons. **W-X** Relative mRNA levels of IL-1β and IL-10 in the mPFC in the LS and LD group. n = 4 mice. Data are represented as mean ± SEM. *$P < 0.05$; **$P < 0.01$, by unpaired Student's t-test was used for **A-D**, **I-M**, **R-X**; two-way ANOVA with Tukey's multiple comparisons test was used for **H and Q**. *Lap* laparotomy, *LS* laparotomy + saline, *LD* laparotomy + dexmedetomidine.

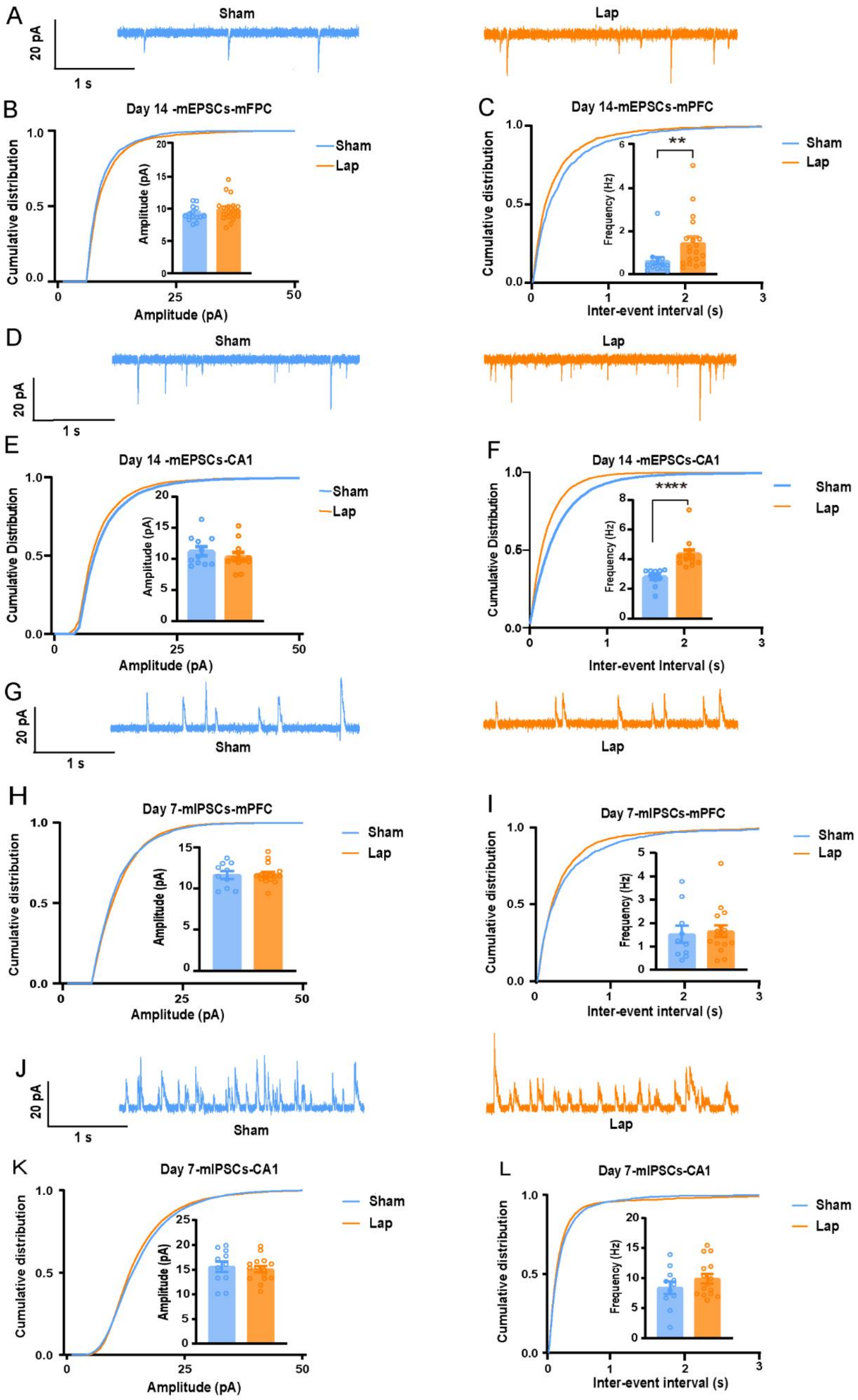
A
Sham
Lap
20 pA
1 s
B
Day 14 -mEPSCs-mFPC
Cumulative distribution
Amplitude (pA)
Sham
Lap
C
Day 14-mEPSCs-mPFC
Cumulative distribution
Frequency (Hz)
Inter-event interval (s)
Sham
Lap
**
D
Sham
Lap
20 pA
1 s
E
Day 14 -mEPSCs-CA1
Cumulative Distribution
Amplitude (pA)
Sham
Lap
F
Day 14 -mEPSCs-CA1
Cumulative Distribution
Frequency (Hz)
Inter-event Interval (s)
Sham
Lap
****
G
20 pA
1 s
Sham
Lap
H
Day 7-mIPSCs-mPFC
Cumulative distribution
Amplitude (pA)
Sham
Lap
I
Day 7-mIPSCs-mPFC
Cumulative distribution
Frequency (Hz)
Inter-event interval (s)
Sham
Lap
J
20 pA
1 s
Sham
Lap
K
Day 7-mIPSCs-CA1
Cumulative distribution
Amplitude (pA)
Sham
Lap
L
Day 7-mIPSCs-CA1
Cumulative distribution
Frequency (Hz)
Inter-event interval (s)
Sham
Lap

Fig. S4 Laparotomy increased pre-synaptic glutamate release 14 days post-surgery without altering inhibitory neurotransmission. **A** Schematic diagram of the mEPSCs traces in mPFC 14 days after laparotomy, n = 14-19 neurons. **B**, **C** Cumulative probability of the frequency and amplitude of mEPSCs 14 days after laparotomy, n = 14-19 neurons. **D** Schematic diagram of the mEPSCs traces in CA1 14 days after laparotomy, n = 11-12 neurons. **E**, **F** Cumulative probability of the frequency and amplitude of mEPSCs 14 days after laparotomy, n = 11-12 neurons. **G** Schematic diagram of the mIPSCs traces in the mPFC 7 days after laparotomy. **H**, **I** Cumulative probability of the frequency and amplitude of mIPSCs in mPFC 7 days after laparotomy, n = 10-16 neurons. **J** Schematic diagram of the mIPSCs traces in CA1 7 days after laparotomy. **K**, **L** Cumulative probability of the frequency and amplitude of mIPSCs in CA1 7 days after laparotomy, n = 11-15 neurons. Data are represented as mean ± SEM. $^{**}P < 0.01$; $^{****}P < 0.0001$, by unpaired Student's t-test was used for **B**-**C**, **E**-**F**, **H**-**I**, **K**-**L**. *Lap* laparotomy.
